# Microbial synthesis of long-chain α-alkenes from methanol by engineering *Pichia pastoris*

**DOI:** 10.1186/s40643-022-00551-1

**Published:** 2022-05-26

**Authors:** Peng Cai, Yunxia Li, Xiaoxin Zhai, Lun Yao, Xiaojun Ma, Lingyun Jia, Yongjin J. Zhou

**Affiliations:** 1 School of Life Science and Biotechnology, Dalian University of Technology, Dalian, 116024 People’s Republic of China; 2 Division of Biotechnology, Dalian Institute of Chemical Physics, Chinese Academy of Sciences, 457 Zhongshan Road, Dalian, 116023 People’s Republic of China; 3 University of Chinese Academy of Sciences, Beijing, 100049 People’s Republic of China; 4 Dalian Key Laboratory of Energy Biotechnology, Dalian Institute of Chemical Physics, Chinese Academy of Sciences, Dalian, 116023 People’s Republic of China; 5 CAS Key Laboratory of Separation Science for Analytical Chemistry, Dalian Institute of Chemical Physics, Chinese Academy of Sciences, Dalian, 116023 People’s Republic of China

**Keywords:** Methylotrophic yeast, *Pichia pastoris*, α-Alkenes, Methanol biorefinery, Cofactor engineering, Peroxisome

## Abstract

α-Alkenes (terminal alkenes) are important fuel and platform chemicals that are mainly produced from petroleum. Microbial synthesis might provide a sustainable approach for α-alkenes. In this work, we engineered the methylotrophic yeast *Pichia pastoris* to produce long-chain (C15:1, C17:1 and C17:2) α-alkenes via a decarboxylation of fatty acids. Combinatorial engineering, including enzyme selection, expression optimization and peroxisomal compartmentalization, enabled the production of 1.6 mg/L α-alkenes from sole methanol. This study represents the first case of α-alkene biosynthesis from methanol and also provides a reference for the construction of methanol microbial cell factories of other high-value chemicals.

**Graphical Abstract:**

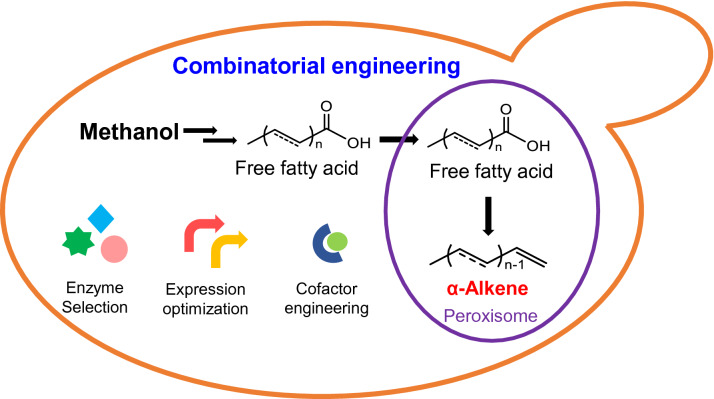

**Supplementary Information:**

The online version contains supplementary material available at 10.1186/s40643-022-00551-1.

## Introduction

**Fig. 1:**
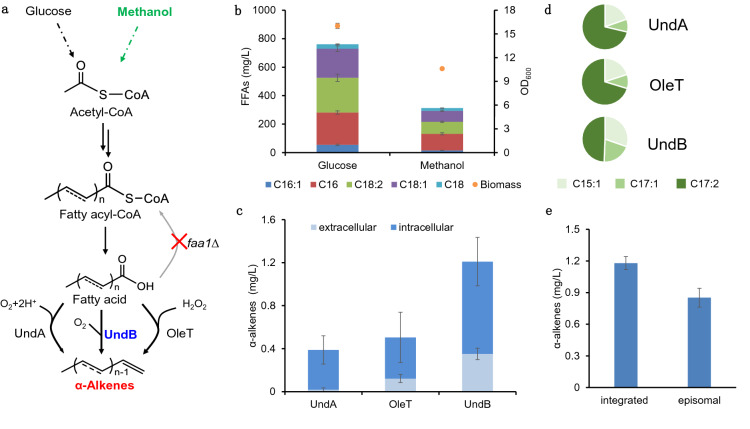
Construction of α-alkenes biosynthetic pathway in *P. pastoris*. **a** Schematic illustration of metabolic pathways engineered for the production of α-alkenes from FFA via three pathways. **b** FFA titers of *faa1*Δ strain in shake flask after 96 h cultivation in 20 g/L glucose medium or 120 h cultivation in 20 g/L methanol medium. **c** α-Alkenes production from three different decarboxylases in glucose medium. **d** α-Alkenes profiles of the engineered strain with expression of different decarboxylases in glucose medium. C15:1, 1-pentadecene; C17:1, 1-heptadecene; C17:2, 1,8-heptadecadiene. **e** Comparison of episomal expression and chromosome integration of *UndB* for α-alkenes production. All data were represented as the mean ± s.d. of three samples

Over the past few decades, there are ongoing attempts to establish the biological processes for sustainable production of chemicals from cheap feedstocks (Zhou et al. 2018b; Sun et al. 2021). α-Alkenes (α-olefin) are important platform chemicals that traditionally used in the production of lubricants and surfactants. In addition, α-alkene is considered as “drop-in" next-generation fuel because of its high energy density and compatibility with existing engine systems (Kang and Nielsen 2017). Free fatty acid (FFA) is an ideal substrate for the biosynthesis of α-alkenes because they have low oxidation state carbon and are widely available. In nature, several enzymes were identified to catalyze the decarboxylation of fatty acids to medium-chain (C8–C12) (Rui et al. 2014) or long-chain (> C12) α-alkenes (Rui et al. 2015; Liu et al. 2014) (Fig. 1a). Some efforts have also been made to introduce these enzymes into model organisms to produce α-alkenes from sugars (Chen et al. 2015; Lee et al. 2018; Liu et al. 2014; Zhou et al. 2018a). However, the titer and yield of α-alkenes produced by microbial cell factories are still too low to meet industrial requirement.

In general, the production process in synthetic biology should consist of cheap raw materials and efficient microbial cell factories. Although glucose has long been used as a fermentation substrate, potential food crisis has prompted people to look for new carbon sources. Methanol, a non-food feedstock, is considered as the core carrier of the next-generation energy system because of its high reducing power per unit carbon, and easy to store and transport (Wang et al. 2020b). In addition, methanol can be massively produced by hydrogenation of carbon dioxide (CO_2_), which is expected to alleviate the greenhouse effect and achieve carbon-neutral production (Liu et al. 2018a, b). Methylotrophic yeast, especially *Pichia pastoris*, is considered to be the ideal host for methanol biomanufacturing because of its good methanol utilization capacity and effective methanol-induced promoters (Schwarzhans et al. 2017; Shi et al. 2020). With the development of synthetic biology tools and gene-editing platforms (Cai et al. 2021, 2019; Gao et al. 2021; Vogl et al. 2016; Weninger et al. 2016; Yan et al. 2022; Yu et al. 2021), a series of chemicals produced from methanol have been reported (Guo et al. 2022; Zhang et al. 2018; Liu et al. 2018a, b; Miao et al. 2021). However, biosynthesis of hydrocarbons, especially α-alkenes, in methylotrophic yeast has not been reported so far.

In the present study, *P. pastoris* was engineered for de novo production of α-alkenes from methanol. The α-alkenes biosynthesis in *P. pastoris* was achieved by enzyme selection and cofactor engineering. Next, production of α-alkenes was further increased by engineering the expression and compartmentalization of the key enzyme UndB. Overall, we successfully realized the biosynthesis of α-alkenes from methanol for the first time, and this work offers a potential approach to achieve carbon neutrality in the production of α-alkenes.

## Materials and methods

### Plasmids, strains and cell cultivation

All the stains and plasmids used in this study are listed and described in Additional file 1: Table S1. *Escherichia coli* DH5α used for plasmid transformation were cultivated at 37 °C in LB medium (10 g/L tryptone, 5 g/L yeast extract, 10 g/L NaCl). 100 μg/mL ampicillin or 50 μg/mL kanamycin was added for plasmid maintenance. *Pichia pastoris* strains for preparation of competent cells were cultivated in YPD medium consisting 20 g/L glucose, 10 g/L yeast extract and 20 g/L peptone. Recombinant strains (chromosomal integration) were selected on YPD plate containing 200 μg/mL G418. Plasmid-carrying strains were screened on synthetic droplet (SD) medium plate containing 20 g/L glucose and 6.7 g/L yeast nitrogen base (YNB). Strains used for real-time PCR were collected from YPM medium consisting 20 g/L methanol, 10 g/L yeast extract and 20 g/L peptone.

Yeast fermentations for production of α-alkenes were carried out in 100-mL shaking flask with a working volume of 20 mL minimal medium (MM), which contains 2.5 g/L (NH_4_)_2_SO_4_, 14.4 g/L KH_2_PO_4_, 0.5 g/L MgSO_4_·7H_2_O, trace metal and vitamin solutions, 20 g/L glucose or 20 g/L methanol, supplemented with 40 mg/L histidine if needed (Cai et al. 2021). The yeast cells were cultivated at 30 °C, 220 rpm (Zhichu Shaker ZQZY-CS8) for 4 days with initial OD_600_ of 0.1 when using glucose as a carbon source, or 5 days with initial OD_600_ of 0.3 when using methanol as a carbon source.

### Genetic manipulation

Gene integration and deletion in *P. pastoris* was also performed by using a CRISPR–Cas9 genome editing system established in our laboratory. All the gRNA expression plasmids were constructed in our previous study (Cai et al. 2021). All the heterologous gene were codon optimized and chemical synthesized at Exsyn-bio Technology Co., Ltd (Shanghai, China) (Additional file 1: Table S2).

Protein expression plasmid pGCAI-UndB was constructed as follows: the plasmid backbone was obtained by PCR amplification with primers ORI-R/Amp-B-R, the replication origin part was obtained with the primers panARS-F2/panARS-B-R, the selection marker was amplified with the primers HIS4p-F2/HIS4T2-R. The above three fragments were assembled with the *UndB* expression cassette by Gibson Assembly^®^ Master Mix according to its manipulating instruction. All the primers are listed in Additional file 1: Table S3.

### Real-time PCR experiment

Since the strains had difficulty growing in MM medium when *UndB* was expressed under strong inducible promoters, engineered strains were cultivated in YPM medium at 30 °C, 220 rpm for 24 h. The total RNA was extracted by RNA simple Total RNA Kit (DP419, TIANGEN, Beijing, China). 1 µg total RNA of each sample was reversely transcribed to cDNA using the PrimeScript® RT reagent Kit (Takara Bio Inc.) according to the manufacturer’s protocol. A two-step PCR reaction was employed by using SYBR® Premix Ex TaqTM II (Takara Bio Inc.). Actin gene was chosen as the endogenous reference gene, and the data analysis was conducted by the method of 2^−∆∆CT^ as described previously (Livak and Schmittgen 2001). Primers are listed in Additional file 1: Table S3, and all strains with three biologically independent parallel samples were adopted to guarantee the reproducibility of all the results.

### Extraction and quantification of α-alkenes

The α-alkenes extraction and quantification were performed as our previous studies (Zhou et al. 2018a) with slight modification. For intracellular α-alkenes analysis, cell pellets were collected from 4 mL glucose cultured cell medium or 8 mL methanol cultured cell medium and subjected to freezer drying for 24 h. Freeze-dried samples were mixed with 4 mL of chloroform–methanol (2:1, v/v) in an extraction glass tube containing 1 mg/L hexadecane as internal standard. The following steps are the same as microwave-based method (Zhou et al. 2018a).

The extracellular α-alkenes were extracted from the supernatant by using *n*-hexane. Two mL hexane containing 1 mg/L hexadecane was added into 4 mL supernatant and the mixtures were shaken for 30 min by using a vortex mixer (1500 rpm). The samples were centrifuged at 2000*g* for 10 min allowing for phase separation and the organic phase was transferred into a new clean extraction glass tube. The extracted sample was then concentrated by drying under vacuum, and resuspended with 200 μL n-hexane. The intracellular and extracellular extraction solvents were analyzed by a GC-FID (Focus GC with a flame ionization detector (FID), Thermo Fisher Scientific) equipped with a Zebron ZB-5MS GUARDIAN capillary column (30 m × 0.25 mm × 0.25 μm, Phenomenex). The GC program for α-alkenes was as follows: initial temperature of 50 °C, hold for 5 min; then ramp to 310 °C at a rate of 10 °C per min and hold for 6 min. The temperature of inlet and detection were kept at 250 °C and 300 °C, respectively. The flow rate of the carrier gas (nitrogen) was set to 1.0 mL per minute, and data were analyzed by using the Xcalibur software.

### Confocal microscopy analysis

For UndB localization analysis, UndB was fused to the N-terminal of two different types of green fluorescent protein (eGFP) (with or without PTS1 signal peptide). The encoding genes were transformed into the yeast strain PC111. The cells were cultivated in minimal media with 20 g/L methanol for 48 h at 30 °C, 220 rpm. 5 μL cell cultures were dropped onto microscope slides and then viewed with a FluoView™ FV1000 confocal microscopy (OLYMPUS, Japan). The excitation wavelength and emission wavelength were 488 nm and 520 nm, respectively.

## Results

### Enzyme selection for α-alkenes biosynthesis in *P. pastoris*

Since fatty acids are the direct precursors for α-alkenes biosynthesis, we first constructed the FFA accumulated strain PC112 by deleting the fatty acyl-CoA synthetase gene (*FAA1*). The engineered strain PC112 produced 728 mg/L FFA in 20 g/L glucose medium and 312 mg/L FFA in 20 g/L methanol medium, respectively (Fig. 1b), meanwhile the parent strains did not accumulate large amounts of fatty acids before *FAA1* deletion (Additional file 1: Fig. S1). The main composition of the product is C16 and C18 fatty acids. At present, three different types of enzymes, OleT_JE_, UndA and UndB were identified to be involved in decarboxylation of fatty acids to α-alkenes (Fig. 1a). Gene Expression cassettes under promoter P_*GAP*_ were constructed and integrated into the PNSII-5 site of *P. pastoris*.

Expression of *PpUndA* from *Pseudomonas putida* F1 (Rui et al. 2014) and *OleT* from *Jeotgalicoccus* (Liu et al. 2014) resulted in the production of 0.4 mg/L and 0.5 mg/L α-alkenes, while expression of *PfUndB* from *Pseudomonas fluorescens* Pf-5 (Rui et al. 2015) enabled a higher production of 1.2 mg/L of α-alkenes in glucose medium (Fig. 1c). *PfUndB* also had the highest α-alkene in methanol medium (Additional file 1: Fig. S2). This result was similar with our previous study in *Saccharomyces cerevisiae* (Zhou et al. 2018a). The products synthesized by UndB are mainly C15:1 (1-pentadecene), C17:1 (1-heptadecene) and C17:2 (1,8-heptadecadiene) with proportions of 31%, 20% and 49%, respectively (Fig. 1d) which suggested that UndB was more suitable to catalyze long-chain fatty acids. In addition, about 29% of the products were secreted out of the cell, which may because UndB is a membrane-bound desaturase-like decarboxylase (Fig. 1d). We also constructed the expression plasmid pGCAI-UndB based on *HIS4* auxotroph marker to further improve the copy number of *UndB* gene. However, the titer of α-alkenes under episomal expression was lower than that of chromosome integration strain, suggesting that chromosome integration was a more stable expression strategy (Fig. 1e).

### Cofactor engineering for enhancing α-alkene biosynthesis

**Fig. 2:**
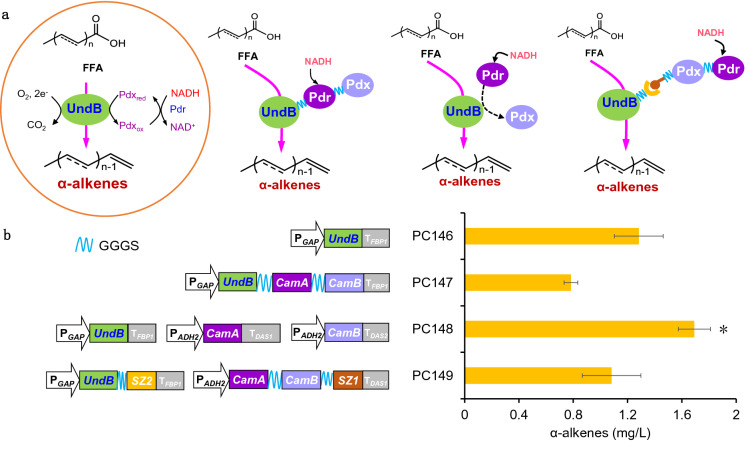
Cofactor engineering for the biosynthesis of α-alkenes. **a** Schematic diagram of different constructed interactions between cofactor and UndB. **b** Total α-alkenes production in engineered strains in shake flask after 96 h cultivation in 20 g/L glucose medium. All data were represented as the mean ± s.d. of three samples. Statistical significance of the different α-alkenes levels in comparison with the control (PC146) was evaluated by using Student’s *t*-test (*P < 0.05; **P < 0.01)

The oxidative decarboxylation of FFA catalyzed by UndB was employing O_2_ as the oxidant and NADH as the electron donor (Fig. 2a) (Rui et al. 2015). We thus tried to construct an efficient electron transfer chain to promote the enzymatic reaction. Our previous study showed that putidaredoxin–putidaredoxin reductase (Pdr/Pdx, encoded by *CamA* and *CamB*, respectively) from *P. putida* resulted in a 108% increment of α-alkene production in *S. cerevisiae*. Therefore, we expressed the *CamA* and *CamB* genes, and optimized the binding mode between cofactor and UndB (Fig. 2a).

We first fused UndB with Pdr/Pdx by a GGGS linker to construct a protein complex for efficient electron channeling. However, this effort resulted in a declining production of α-alkene, which might due to the incorrect folding of fused protein that decreased the catalytic efficiency of the enzymes. Similar results have been reported in our previous study (Zhou et al. 2018a). Alternatively, separate expression of *UndB* and *CamA*/*B* (strain PC148) produced 1.7 mg/L α-alkenes, a 33% improvement compared with the control strain PC146 (Fig. 2b). Previous studies showed that that pairs of heteroassociating coiled peptides can help to construct protein interactions through non-covalent bonds (Reinke et al. 2010; Thompson et al. 2012). We thus designed a protein adsorption strategy based on affinity peptides to facilitate the electron channel. We fused a pair of synthetic coiled-coil zippers (SYNZIP1 and SYNZIP2; hereafter referred to as SZ1 and SZ2) to C terminus of UndB and cofactor protein, respectively (Klaus et al. 2019; Thomik et al. 2017). However, this design resulted in a slight decrease in α-alkene production, possibly due to the addition of sequences and their location (N-terminal or C-terminal) corresponding to different engineering enzyme activities (Thomik et al. 2017).

### Production of α-alkene by using methanol as sole carbon source

**Fig. 3:**
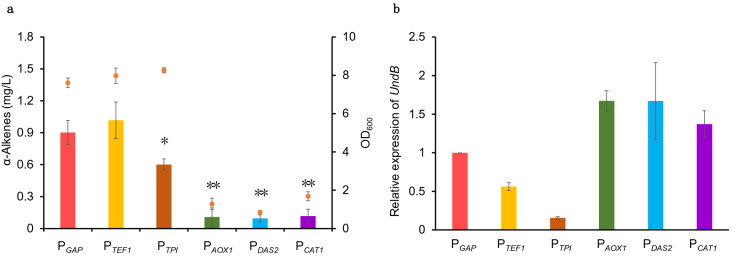
Fermentation of α-alkenes from methanol as sole carbon resource. **a** Total α-alkenes production in corresponding strains in shake flask after 120 h cultivation in 20 g/L methanol medium. **b** The relative expression levels of *UndB* under different promoters. The housekeeping gene actin was chosen as baseline for quantification of expression level. Relative expression levels of *UndB* were calculated by normalized the expression level under P_*GAP*_. All data were represented as the mean ± s.d. of three samples. Statistical significance of the different α-alkenes levels in comparison with the control (P_*GAP*_) was evaluated using Student’s *t* test (*P < 0.05; **P < 0.01)

Methanol-induced promoters such as P_*AOX1*_, have long been used for the production of recombinant proteins (Vogl et al. 2016; Yang and Zhang 2018). Our previous study also suggested that constitutive promoters can continuously drive the expression of proteins in methanol medium (Cai et al. 2021). Therefore, three methanol-induced promoters (P_*AOX1*_, P_*DAS2*_ and P_*CAT1*_) and three constitutive promoters (P_*GAP*_, P_*TEF1*_ and P_*TPI*_) were selected to express the *UndB* in methanol culture (Fig. 3a). Expression of *UndB* by methanol-induced promoters seriously hindered cell growth, and only trace amounts of olefin were detected (Fig. 3a). On the contrary, *UndB* expression under constitutive promoters had better growth than that of the methanol-induced promoter, and the highest α-alkene titer was 1 mg/L in methanol medium with a cell density (OD_600_) of 8.0 when *UndB* was expressed under P_*TEF1*_ promoter (Fig. 3a). Our previous studies showed that the strength of these three methanol-induced promoters was much higher than that of the constitutive promoters (Cai et al. 2021). Consistently, real-time PCR analysis showed that these methanol-induced promoters had significantly higher transcription of *UndB* than constitutive promoters (Fig. 3b). Since UndB is a membrane localization protein, its high expression may affect cell membrane fluidity and bring stress on cell function. Again, construction an affinity adsorption of cofactors to *UndB* resulted a lower α-alkene production in methanol medium (Additional file 1: Fig. S3).

### Production of α-alkenes in peroxisome from methanol

**Fig. 4:**
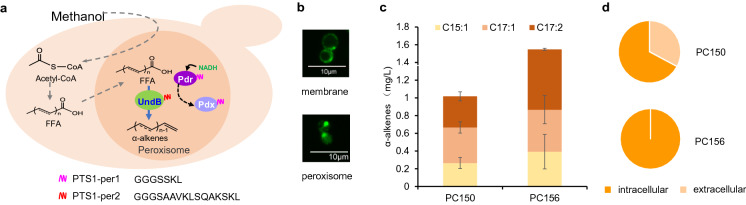
Peroxisomal compartmentalization improved α-alkenes production in *P. pastoris*. **a** Schematic view of the metabolic pathway. **b** Confocal fluorescence microscopy analysis of PC111 that carried an *UndB*-*eGFP* (membrane) or *UndB*-*eGFP*-*Per2* (peroxisome) fusion gene. **c** α-Alkenes titers from the engineered strains. C15:1, 1-pentadecene; C17:1, 1-heptadecene; C17:2, 1,8-heptadecadiene. **d** The distribution of α-alkenes from methanol. All data were represented as the mean ± s.d. of three samples

In methylotrophic yeast, the methanol metabolism occurs in peroxisome (Hammer and Avalos 2017). Previous reports have demonstrated the advantages of peroxisomal targeting for enhancing the biosynthesis of fatty acid derivatives (Sheng et al. 2016; Zhou et al. 2016). We here thus targeted the UndB and cofactor proteins to the peroxisome with the peroxisomal targeting signals (Fig. 4a). Fluorescence microscopy showed correct localization (Fig. 4b). The engineered strain PC156 produced 1.6 mg/L α-alkenes from methanol, a 52.1% improvement compared to the strain PC150 that containing the cytosolic pathway (Fig. 4c). The composition of olefins in peroxisome was consistent with that in cytoplasm. Interestingly, there was no detectable extracellular α-alkenes with the peroxisomal compartmentalized pathway (Fig. 4d). This result may be due to the existence of local hydrophobic regions in the peroxisome, is conducive to the storage of long-chain olefins.

## Discussion

Though short chain olefins (C2–C4) were synthesized easily from methanol by chemical process (Tian et al. 2015), it is still difficult to produce long-chain alkenes chemically. Construction of microbial cell factory provides another chance for producing long-chain α-alkenes from methanol since bio-catalysis has high selectivity and specificity. We here showed construction of yeast cell factory for synthesis of odd long-chain alkenes from sole methanol.

The α-alkenes synthesis with UndB in *P. pastoris* was higher than that of UndA and OleT (Fig. 1c), which may be due to substrate selectivity of the enzyme. Previous studies have shown that UndA and OleT may be more likely to catalyze short- and medium-chain fatty acids (Dennig et al. 2015; Rui et al. 2014), while UndB can catalyze long-chain fatty acids which mainly accumulated in *P. pastoris* (Zhou et al. 2018a). Electron transfer is also crucial for decarboxylation reaction besides decarboxylase. The H_2_O_2_-independent cofactor system Fdr/Fdx avoided the peroxidation damage of cells and improved the efficiency of decarboxylation reaction (Kang and Nielsen 2017; Zhou et al. 2018a). In addition, the regulation of methanol metabolism needs to be fully considered, and the expression of the membrane enzymes UndB should be carefully tuned for alkene synthesis to avoid the metabolic stress in methanol medium. The current low titer was mainly attributed to the low catalytic efficiency of decarboxylation. Therefore, protein engineering including rational design (Matthews et al. 2017; Liu and Li, 2020) and directed evolution (Wang et al. 2020a) might be helpful to improve catalytic efficiency. In addition, discovering efficient enzymes (Jiang et al. 2019) from natural resources can be also cosidered as a feasible approach.

In summary, we realized the production of α-alkenes by using methanol as sole carbon resource. Though the titer was low, this study clearly showed that methanol can be used as a feedstock for future biomanufacturing.

### Supplementary Information

**Additional file 1**: **Figure S1. **Fatty acid production in wild-type *P. pastoris* from methanol. All data were represented as the mean ± s.d. of three samples. **Figure S2. **α-Alkenes production from three different decarboxylases in methanol medium. All data were represented as the mean ± s.d. of three samples. **Figure S3. **Cofactor engineering for the biosynthesis of α-alkenes in methanol medium. All data were represented as the mean ± s.d. of three samples. Statistical significance of the different α-alkene levels in comparison with the control (PC146) was evaluated using Student’s *t*-test (*, *P* < 0.05; **, *P* < 0.01). **Table S1.** Strains and plasmids used in this study**. Table S2.** Codon optimized sequence in this study. **Table S3. **Primers used in this study.

## Data Availability

All data generated during this study are included in this article.
