# Supplementary material for: Microbial synthesis of long-chain α-alkenes from methanol by engineering Pichia pastoris
Source: Bioresour Bioprocess. 2022 May 26;9(1):58. doi: 10.1186/s40643-022-00551-1 (PMC10991524; doi:10.1186/s40643-022-00551-1)
Supplement: Supplementary file 1 — Additional file 1: Figure S1. Fatty acid production in wild-type P. pastoris from methanol. All data were represented as the mean ± s.d. of three samples. Figure S2. α-Alkenes production from three different decarboxylases in methanol medium. All data were represented as the mean ± s.d. of three samples. Figure S3. Cofactor engineering for the biosynthesis of α-alkenes in methanol medium. All data were represented as the mean ± s.d. of three samples. Statistical significance of the different α-alkene levels in comparison with the control (PC146) was evaluated using Student’s t-test (*, P < 0.05; **, P < 0.01). Table S1. Strains and plasmids used in this study. Table S2. Codon optimized sequence in this study. Table S3. Primers used in this study. [file 40643_2022_551_MOESM1_ESM.docx]

Additional file 1 for

**Microbial synthesis of** **long chain α-alkenes from methanol by engineering *Pichia pastoris***

Peng Cai ^1,2,3^, Yunxia Li ^2,5^, Xiaoxin Zhai ^2,5^, Lun Yao ^4,5^, Xiaojun Ma ^4,5^, Lingyun Jia ^1^, Yongjin J. Zhou ^2,4,5^*

^1^School of Life Science and Biotechnology, Dalian University of Technology, Dalian 116024, P. R. China

^2^Division of Biotechnology, Dalian Institute of Chemical Physics, Chinese Academy of Sciences, 457 Zhongshan Road, Dalian 116023, P. R. China

^3^University of Chinese Academy of Sciences, Beijing 100049, P. R. China

^4^Dalian Key Laboratory of Energy Biotechnology, Dalian Institute of Chemical Physics, Chinese Academy of Sciences, Dalian 116023, P. R. China

^5^CAS Key Laboratory of Separation Science for Analytical Chemistry, Dalian Institute of Chemical Physics, Chinese Academy of Sciences, Dalian 116023, P. R. China

*Correspondence to: Yongjin J. Zhou. +84-411-84771060 Email: [zhouyongjin@dicp.ac.cn](mailto:zhouyongjin@dicp.ac.cn)

**Figure S1. Fatty acid production in wild-type *P. pastoris* from methanol**. All data was represented as the mean ± s.d. of three samples.

**Figure S2. α-Alkenes production from three different decarboxylases in methanol medium.** All data was represented as the mean ± s.d. of three samples.

**Figure S3. Cofactor engineering for the biosynthesis of α-alkenes in methanol medium.** All data was represented as the mean ± s.d. of three samples. Statistical significance of the different α-alkenes levels in comparison with the control (PC146) was evaluated using Student’s *t*-test (*, *P* < 0.05; **, *P* < 0.01).

**Table S1. Strains and plasmids used in this study**

| Strain | Genotype | | Source | |
| --- | --- | --- | --- | --- |
| DH5α | F-, φ80d/lacZΔM15, Δ(*lacZYA-argF*)U169, *deoR*, *recA1*, *endA1*, *hsdR17*(rk-, mk+), *phoA*, *supE44*, λ-, *thi-1*, *gyrA96*, *relA1* | | | Takara |
| PC111 | GS115, *his4Δ::*P*_GAP_*-*PpRAD52*-T*_AOX1_*, *PNSI-2::*P_GAP_*-hCas9-*T*_DAS1_* | | | [[1](#_ENREF_1)] |
| PC112 | GS115, *his4Δ*::P*_GAP_*-*PpRAD52*-T*_AOX1_, PNSI-3::*P_GAP_*-hCas9-*T*_DAS1_*, *faa1*Δ =[PC111]+(*faa1Δ*) | | | This study |
| PC142 | GS115, *his4Δ*::P*_GAP_*-*PpRAD52*-T*_AOX1_, PNSI-3::*P_GAP_*-hCas9-*T*_DAS1_*, *faa1*Δ, *PNSII-3*::P*_ADH2_*-*PpCamA*-T*_DAS1_*, *PNSII-7*::P*_ADH2_*-*PpCamB*-T*_DAS2_* =[PC112]+( *PNSII-3*::P*_ADH2_*-*PpCamA*-T*_DAS1_*, *PNSII-7*::P*_ADH2_*-*PpCamB*-T*_DAS2_*) | | | This study |
| PC143 | GS115, *his4Δ*::P*_GAP_*-*PpRAD52*-T*_AOX1_, PNSI-3::*P_GAP_*-hCas9-*T*_DAS1_*, *faa1*Δ, *PNSII-5*::P*_GAP_*-*PpUndA*-T*_FBP1_* =[PC112]+(*PNSII-5*::P*_GAP_*-*PpUndA*-T*_FBP1_*) | | | This study |
| PC144 | GS115, *his4Δ*::P*_GAP_*-*PpRAD52*-T*_AOX1_, PNSI-3::*P_GAP_*-hCas9-*T*_DAS1_*, *faa1*Δ, *PNSII-5*::P*_GAP_*-*JeOleT*-T*_FBP1_* =[PC112]+(*PNSII-5*::P*_GAP_*- *JeOleT*-T*_FBP1_*) | | | This study |
| PC145 | GS115, *his4Δ*::P*_GAP_*-*PpRAD52*-T*_AOX1_, PNSI-3::*P_GAP_*-hCas9-*T*_DAS1_*, *faa1*Δ, pGCAI-P*_GAP_*-*PfUndB*-T*_FBP1_* =[PC112]+( pGCAI-P*_GAP_*-*PfUndB*-T*_FBP1_*) | | | This study |
| PC146 | GS115, *his4Δ*::P*_GAP_*-*PpRAD52*-T*_AOX1_, PNSI-3::*P_GAP_*-hCas9-*T*_DAS1_*, *faa1*Δ, *PNSII-5*::P*_GAP_*-*PfUndB*-T*_FBP1_* =[PC112]+(*PNSII-5*::P*_GAP_*-*PfUndB*-T*_FBP1_*) | | | This study |
| PC147 | GS115, *his4Δ*::P*_GAP_*-*PpRAD52*-T*_AOX1_, PNSI-3::*P_GAP_*-hCas9-*T*_DAS1_*, *faa1*Δ, *PNSII-5*::P*_GAP_*-*PfUndB*~*PpCamA*~*CamB*-T*_FBP1_* =[PC112]+(*PNSII-5*::P*_GAP_*-*PfUndB*~*PpCamA*~*CamB*-T*_FBP1_*) | | | This study |
| PC148 | GS115, *his4Δ*::P*_GAP_*-*PpRAD52*-T*_AOX1_, PNSI-3::*P_GAP_*-hCas9-*T*_DAS1_*, *faa1*Δ, *PNSII-3*::P*_ADH2_*-*PpCamA*-T*_DAS1_*, *PNSII-7*::P*_ADH2_*-*PpCamB*-T*_DAS2_*, *PNSII-5*::P*_GAP_*-*PfUndB*-T*_FBP1_*  =[PC142]+(*PNSII-5*::P*_GAP_*-*PpUndB*-T*_FBP1_*) | | | This study |
| PC149 | GS115, *his4Δ*::P*_GAP_*-*PpRAD52*-T*_AOX1_, PNSI-3::*P_GAP_*-hCas9-*T*_DAS1_*, *faa1*Δ, *PNSII-5*::P*_GAP_*-*PfUndB*~SZ2-T*_FBP1_ PNSII-3*::P*_ADH2_*-*PpCamA*~*PpCamB*~SZ1-T*_DAS1_*  =[PC112]+(*PNSII-5*::P*_GAP_*-*PfUndB*~SZ2-T*_FBP1_ PNSII-3*::P*_ADH2_*-*PpCamA*~*PpCamB*~SZ1-T*_DAS1_*) | | | This study |
| PC150 | GS115, *his4Δ*::P*_GAP_*-*PpRAD52*-T*_AOX1_, PNSI-3::*P_GAP_*-hCas9-*T*_DAS1_*, *faa1*Δ, *PNSII-3*::P*_ADH2_*-*PpCamA*-T*_DAS1_*, *PNSII-7*::P*_ADH2_*-*PpCamB*-T*_DAS2_*, *PNSII-5*::P*_TEF1_*-*PfUndB*-T*_FBP1_*  =[PC142]+(*PNSII-5*::P*_TEF1_*-*PpUndB*-T*_FBP1_*) | | | This study |
| PC156 | GS115, *his4Δ*::P*_GAP_*-*PpRAD52*-T*_AOX1_, PNSI-3::*P_GAP_*-hCas9-*T*_DAS1_*, *faa1*Δ, *PNSII-3*::P*_ADH2_*-*PpCamA*~PTS1-T*_DAS1_*, *PNSII-7*::P*_ADH2_*-*PpCamB*~PTS1-T*_DAS2_*, *PNSII-5*::P*_TEF1_*-*PfUndB*~PTS2-T*_FBP1_*  =[PC112]+(*PNSII-3*::P*_ADH2_*-*PpCamA*~PTS1-T*_DAS1_*, *PNSII-7*::P*_ADH2_*-*PpCamB*~PTS1-T*_DAS2_*, *PNSII-5*::P*_TEF1_*-*PfUndB*~PTS2-T*_FBP1_*) | | | This study |
| **Plasmid name** | | **Relevant characteristics** | **Source** | |
| pPICZ-Cas9-gFAA1 | | panARS, *Ble^R^*, P*_HTX1_*-Cas9-T*_DAS1_*, P*_HTX1_*-gFAA1-T*_AOX1_* | | [[1](#_ENREF_1)] |
| pCAI-gPNSII-3 | | panARS, *KanMX*, P*_HTX1_*-gPNSII-3-T*_AOX1_* | | [[1](#_ENREF_1)] |
| pCAI-gPNSII-5 | | panARS, *KanMX*, P*_HTX1_*-gPNSII-5-T*_AOX1_* | | [[1](#_ENREF_1)] |
| pCAI-gPNSII-7 | | panARS, *KanMX*, P*_HTX1_*-gPNSII-7-T*_AOX1_* | | [[1](#_ENREF_1)] |

**Table S2. Codon optimized sequence in this study**

| NO. | Name/  Length (bp) | Sequence (5'-3') | Source |
| --- | --- | --- | --- |
| 1 | CamA-KpOpt  1269bp | ATGAACGCCAACGACAATGTTGTCATCGTCGGTACCGGACTTGCCGGAGTTGAAGTTGCCTTCGGTTTGAGGGCTTCTGGTTGGGAGGGTAACATTAGATTGGTTGGAGACGCCACTGTTATCCCTCACCACTTGCCTCCTCTTTCTAAGGCTTACTTGGCTGGTAAGGCCACTGCCGAGTCTCTTTACCTTAGGACCCCAGACGCCTACGCCGCCCAGAACATCCAACTTTTGGGAGGTACCCAAGTTACCGCTATCAATAGAGATAGGCAGCAAGTTATCTTGAGTGACGGTAGAGCCTTGGATTACGATAGACTTGTCCTTGCCACCGGAGGAAGACCAAGACCTTTGCCAGTCGCCTCTGGAGCTGTCGGAAAGGCTAACAACTTTAGATACCTTAGAACCTTGGAGGACGCCGAGTGTATCAGAAGGCAGTTGATCGCCGACAATAGATTGGTTGTCATCGGAGGTGGATACATCGGATTGGAAGTCGCCGCCACCGCCATCAAAGCCAACATGCACGTCACCCTTCTTGACACCGCTGCCAGAGTCCTTGAAAGGGTTACTGCCCCTCCAGTCTCTGCCTTTTACGAGCACTTGCATAGGGAGGCTGGAGTTGATATTAGAACCGGTACCCAAGTCTGCGGATTCGAGATGTCTACCGACCAGCAGAAGGTTACCGCTGTTCTTTGCGAGGACGGTACTAGGTTGCCAGCTGACTTGGTCATCGCCGGTATCGGTCTTATCCCAAACTGCGAACTTGCCTCTGCTGCCGGTTTGCAAGTTGACAACGGAATCGTCATCAACGAACACATGCAGACCTCTGACCCTCTTATTATGGCCGTTGGTGACTGCGCTAGGTTCCATTCTCAGTTGTACGATAGATGGGTTAGAATTGAGTCTGTCCCTAACGCCCTTGAACAAGCTAGAAAGATCGCCGCTATCCTTTGCGGTAAGGTCCCTAGGGATGAAGCTGCCCCTTGGTTCTGGAGTGATCAGTATGAAATCGGATTGAAGATGGTCGGTCTTAGTGAGGGATACGATAGGATCATCGTTAGAGGTTCTCTTGCCCAACCAGACTTTTCTGTCTTCTACTTGCAAGGAGACAGAGTCCTTGCTGTCGATACCGTCAATAGACCAGTTGAGTTCAACCAATCTAAGCAAATCATCACCGATAGATTGCCAGTTGAACCAAACTTGCTTGGTGACGAGAGTGTCCCACTTAAGGAGATCATCGCCGCTGCTAAGGCCGAACTTTCTAGTGCCTGA | This study |
| 2 | CamB-KpOpt  324bp | ATGTCTAAGGTCGTCTACGTCTCTCACGACGGTACTAGGAGAGAGTTGGATGTCGCTGACGGAGTCTCTCTTATGCAAGCTGCCGTCTCTAACGGAATCTACGACATCGTCGGTGACTGTGGAGGTAGTGCTTCTTGCGCTACTTGCCACGTCTACGTCAACGAGGCCTTCACCGATAAGGTTCCAGCCGCTAACGAGAGGGAGATCGGAATGTTGGAGTGTGTCACCGCCGAGTTGAAGCCAAACTCTAGGCTTTGCTGCCAGATCATCATGACCCCAGAGTTGGACGGTATCGTCGTCGATGTTCCAGATAGGCAGTGGTGA | This study |
| 3. | PfUndB-KpOpt  1074 bp | ATGCAAGGAATCTCTGCTAGTCCAGAGAGGATGAACGCCCAGCAAAGAGCCGCCCACGTTAGACAAGTTGTCTTGGCCAGAGGAGACGAGCTTAGGAGGAGGTTCCCTCTTTTGAGACACCAAGATGCCCTTGGTGCTGGAATCTTGGCCTTCGCCTTGTCTGGAATGTTGGGATCTGCCCTTTTGTACGTCACCGGTCATCTTGCTTGGTGGGCTTGCTTGTTGCTTAACGCCTTCTTCGCCTCTCTTACCCACGAGTTGGAGCATGACCTTATTCATTCTATGTACTTTAGAAAGCAGAGGTTGCCTCATAACCTTATGCTTGGATTGGTTTGGTTGGCTAGACCATCTACTATCAACCCATGGGTTAGAAGGCACCTTCATCTTAACCACCACAAGGTCAGTGGATCTGAGAGTGACATCGAGGAGAGAGCTATCACCAATGGTGAGCCATGGGGAATCGCTAGACTTTTGATGGTCGGTGACAACATGATGGCTGCCTTCATTAGACTTCTTAGGGCTCCCGGAGCTAGGAGAAAGTTGGGAATCCTTGTTAGAACCTTGGCCGTTTATGCCCCTTTGGCCTTGTTGCATTGGGGAGCTTGGTACGTCTTCCTTGGATTCCACGGTGCTAACGGAGTCGCCGCCTTGCTTGGATCTCCAATCCAGTGGTCTCAAGATACCGCCAGTTTGATGCATTACGTCGACATCGCCGTCGTCGTCATCATCGGACCAAACGTTTTGAGAACTTTCTGCTTGCATTTTGTCTCTTCTAACATGCATTACTATGGAGACATCGAGCCCGGAAACGTCATCCAGCAGACCCAAGTTCTTAACCCATGGTGGATGTGGCCACTTCAAGCCTTCTGCTGCAACTTCGGTTCTACCCATGGAATCCACCACTTCGTCGTTAGAGAGCCTTTCTACATTAGACAGATGACTGCCTCTGTCGCTCATAAGGTCATGGCCGAGATGGGTGTTAGATTCAATGACTTCGGTACCTTTGCTAGAGCTAATAGATTCACTAGACAAGAAAGGGAAGCCATGCAACCAGCCCACAATGCTAGAGCCTGA | This study |
| 4 | PpUndA-KpOpt  816 | ATGGAAATCACTAGAATTAAGGAGTTGAAGGTCATCGACGCCTTCGTCAGAATCGGACCACTTATGGATCCAGCTAGTTATCCACAGTGGGCCCAGCAATTGATCGAGGACTGCAGAGAATCTAAGAGGAGGGTTGTCGAGCACGAGTTCTACGCTAGATTGAGGGACGGACAGCTTAAGCAGTCTACCATTAGACAGTACCTTATCGGTGGATGGCCAGTCGTCGAGCAGTTCAGTCTTTATATGGCCCACAACCTTACCAAGACTAGATACGGTAGACACCAAGGAGAAGATATGGCTAGAAGGTGGCTTATGAGGAACATTAGAGTCGAGCTTAACCACGCCGACTACTGGGTCAACTGGTGTCAAGCTCATGGTGTTCACCTTCACGAGCTTCAAGCTCAAGAAGTCCCACCAGAGCTTAACGGACTTAACGACTGGTGCTGGAGAGTTTGCGCCACCGAAAACTTGGCCATCTCTATGGCCGCCACTAACTACGCTATCGAGGGAGCTACTGGAGAGTGGTCTGCTGTCGTCTGCAGTACCGATACCTATGCCCAAGGATTCCCAGAAGAAGGTAGGAAGAGGGCCATGAAGTGGCTTAAGATGCACGCTCAGTACGACGATGCCCACCCATGGGAGGCTTTGGAAATCATCTGCACCCTTGCCGGTGAAAACCCTACCTTGGGTTTGAGAACCGAGCTTAGGAGGGCCATCTGCAAGTCTTACGACTGCATGTTTCTTTTCCTTGAAAGGTGCATGCAGTTGGAGGGAAGGCAACAAGGAAGGATGAGACCAGCTCTTGCTGCTGGATGA | This study |
| 5 | JeOleT-KpOpt  1269 bp | ATGGCTACTTTGAAAAGAGATAAAGGATTGGACAATACCCTTAAGGTTCTTAAACAAGGATATCTTTATACTACCAACCAGAGGAATAGATTGAACACCTCTGTCTTCCAGACCAAAGCCTTGGGAGGAAAGCCATTCGTCGTCGTCACCGGAAAGGAAGGTGCCGAGATGTTCTACAATAACGACGTCGTCCAAAGGGAGGGAATGTTGCCTAAGAGGATCGTCAACACCCTTTTCGGAAAGGGAGCTATCCACACCGTCGATGGTAAGAAGCACGTCGATAGGAAGGCTCTTTTCATGAGTCTTATGACCGAAGGAAACTTGAACTACGTCAGAGAGCTTACTAGAACCTTGTGGCACGCCAATACCCAGAGGATGGAGTCTATGGACGAAGTCAACATCTACAGAGAGAGTATCGTTTTGTTGACTAAAGTCGGAACTAGATGGGCTGGAGTTCAAGCTCCTCCAGAAGATATCGAAAGGATCGCCACCGACATGGACATCATGATCGATAGTTTCAGAGCCCTTGGAGGTGCCTTCAAGGGATACAAGGCCTCTAAGGAGGCTAGAAGGAGAGTTGAGGACTGGTTGGAGGAGCAAATCATCGAGACTAGGAAGGGAAACATCCACCCTCCAGAAGGAACCGCTTTGTACGAGTTCGCCCACTGGGAGGATTACCTTGGTAACCCAATGGACTCTAGGACTTGTGCCATCGACCTTATGAACACCTTCAGACCTCTTATTGCCATTAATAGGTTCGTCAGTTTCGGATTGCACGCCATGAACGAGAACCCTATTACTAGGGAAAAGATCAAGTCTGAGCCAGACTACGCCTACAAGTTCGCTCAAGAAGTTAGAAGGTACTACCCATTCGTCCCATTCTTGCCCGGAAAGGCCAAGGTCGACATTGACTTCCAAGGAGTTACCATCCCAGCTGGAGTTGGATTGGCCTTGGACGTCTACGGTACCACCCACGATGAGAGTCTTTGGGACGACCCTAACGAGTTCAGACCAGAGAGATTCGAGACTTGGGACGGATCTCCTTTCGACCTTATTCCACAAGGAGGTGGAGACTACTGGACCAACCATAGATGCGCCGGAGAATGGATCACCGTTATCATCATGGAAGAAACCATGAAATACTTTGCCGAGAAAATCACCTACGACGTTCCAGAGCAAGATCTTGAGGTCGACCTTAACTCTATCCCCGGATACGTCAAGAGTGGTTTCGTCATCAAGAACGTTAGAGAGGTCGTTGACAGAACTTGA | This study |

**Table S3. Primers used in this study.**

| Primer  No. | Name | Sequence (5’‐3’) |
| --- | --- | --- |
| P1 | CamAopt-ADH2p-F | ACTAGCTTTTATCTTATTTACTTTACGAAAATGAACGCCAACGACAATGTTG |
| P2 | CamAopt-DAS1t-R | CTCCTAACTAAAACTGTAAAGACTTCCCGTTCAGGCACTAGAAAGTTCGGCC |
| P3 | CamBopt-ADH2p-F | ACTAGCTTTTATCTTATTTACTTTACGAAAATGTCTAAGGTCGTCTACGTCTCTCAC |
| P4 | CamBopt-DAS2t-R | AACTACTAACCCGTTAGTGGCCAAATCTACTCACCACTGCCTATCTGGAACATCG |
| P5 | PpUndAopt-GAPp-F | TTCAATCAATTGAACAACTATCAAAACACAATGGAAATCACTAGAATTAAGGAGTTGAAGGTC |
| P6 | PpUndAopt-FBP1t-R | AAATCTCGGAAACAGTGCCAATCGAACGCATCATCCAGCAGCAAGAGCTGG |
| P7 | OleTopt-GAPp-F | TTCAATCAATTGAACAACTATCAAAACACAATGGCTACTTTGAAAAGAGATAAAGGATTGGA |
| P8 | OleTopt-FBP1t-R | AAATCTCGGAAACAGTGCCAATCGAACGCATCAAGTTCTGTCAACGACCTCTCTAACG |
| P9 | PfUndBopt-GAPp-F | ATTTCAATCAATTGAACAACTATCAAAACACAATGCAAGGAATCTCTGCTAGTCCAG |
| P10 | PfUndBopt-FBP1t-R | AAATCTCGGAAACAGTGCCAATCGAACGCATCAGGCTCTAGCATTGTGGGC |
| P11 | CamAper1opt-DAS1t-R | CTCCTAACTAAAACTGTAAAGACTTCCCGTTCACAACTTAGAAGAACCTCCTCCG |
| P12 | CamBper1opt-DAS2t-R | AACTACTAACCCGTTAGTGGCCAAATCTACTCACAACTTAGAAGATCCTCCTCCCCA |
| P13 | PfUndBper2opt-FBP1t-R | AAATCTCGGAAACAGTGCCAATCGAACGCATCACAATTTACTTTTGGCTTGACTAAGCT |
| P14 | GAPp-SpeI-F | GGAAGTAAGATGACACTAGTTTTTTGTAGAAATGTCTTGGTGTCCTCG |
| P15 | Amp-B-R | ACTAGTGTCATCTTACTTCCCGTCTTAGAACTGCCATGTAGCTGG |
| P16 | ORI-R | ACGCGTGTACGCATGTAACATTATACTGAAAACCTTGCTTGAGAAGG |
| P17 | panARS-F2 | CTCGAGGATCCTCAACATCTTTGGATAATATCAGAATGAGAAAG |
| P18 | panARS-B-R | CCGCGGGCGGTAGTAAAATCTCTAGATAGTGCTGATTATGATTTGACGTTTATATACA |
| P19 | HIS4T2-R | ATGTTACATGCGTACACGCGTGATCTATCGAATCTAAATGTAAGTTAAAATCTCTAAAT |
| P20 | HIS4p-F2 | ATATTATCCAAAGATGTTGAGGATCCTCGAGGATCTCCTGATGACTGACTCACTG |
| P21 | FBP1t-SacII-R2 | ACTATCTAGAGATTTTACTACCGCCCGCGGCGCGGAACCTTACTTTTCTATTATCCCTA |
| P22 | ACT1-RTpcr-F | ACCCACGTTGTCCCAATTTATG |
| P23 | ACT1-RTpcr-R | CTCCTTGATGTCACGGACGATT |
| P24 | UndB-RT-F | GAGGATGAACGCCCAGCAAAGAG |
| P25 | UndB-RT-R | AAGCAACAAGCAAGCCCACCAAG |

Reference

1. Cai P, Duan X, Wu X, Gao L, Ye M, Zhou YJ: Recombination machinery engineering facilitates metabolic engineering of the industrial yeast *Pichia pastoris***.** *Nucleic Acids Res* 2021, **49:**7791-7805.
